# Classifying oscillatory brain activity associated with Indian *Rasa*s using network metrics

**DOI:** 10.1186/s40708-022-00163-7

**Published:** 2022-07-15

**Authors:** Pankaj Pandey, Richa Tripathi, Krishna Prasad Miyapuram

**Affiliations:** 1 Computer Science and Engineering, Indian Institute of Technology Gandhinagar, 382355 Gandhinagar, India; 2 Center for Advanced Systems Understanding (CASUS), Helmholtz-Zentrum Dresden-Rossendorf, Görlitz, Germany; 3 Centre for Cognitive and Brain Sciences, Indian Institute of Technology Gandhinagar, 382355 Gandhinagar, India

**Keywords:** EEG, Emotion, Classification, *Natyashastra*, *Rasas*, Movie clips, Random Forest, wPLI, Graph theory

## Abstract

Neural signatures for the western classification of emotions have been widely discussed in the literature. The ancient Indian treatise on performing arts known as *Natyashastra* categorizes emotions into nine classes, known as *Rasa*s. *Rasa*—as opposed to a pure emotion—is defined as a superposition of certain transitory, dominant, and temperamental emotional states. Although *Rasa*s have been widely discussed in the text, dedicated brain imaging studies have not been conducted in their research. Our study examines the neural oscillations, recorded through electroencephalography (EEG) imaging, that are elicited while experiencing emotional states corresponding to *Rasa*s. We identify differences among them using network-based functional connectivity metrics in five different frequency bands. Further, Random Forest models are trained on the extracted network features, and we present our findings based on classifier predictions. We observe slow (delta) and fast brain waves (beta and gamma) exhibited the maximum discriminating features between *Rasa*s, whereas alpha and theta bands showed fewer distinguishable pairs. Out of nine *Rasa*s, Sringaram (love), Bibhatsam (odious), and Bhayanakam (terror) were distinguishable from other *Rasa*s the most across frequency bands. On the scale of most network metrics, Raudram (rage) and Sringaram are on the extremes, which also resulted in their good classification accuracy of 95%. This is reminiscent of the circumplex model where anger and contentment/happiness are on extremes on the pleasant scale. Interestingly, our results are consistent with the previous studies which highlight the significant role of higher frequency oscillations in the classification of emotions, in contrast to the alpha band that has shows non-significant differences across emotions. This research contributes to one of the first attempts to investigate the neural correlates of *Rasa*s. Therefore, the results of this study can potentially guide the explorations into the entrainment of brain oscillations between performers and viewers, which can further lead to better performances and viewer experience.

## Introduction

Our emotions affect our daily lives in many ways and they contribute to cognitive processes such as perception, attention, and decision-making. Films engage viewers through experiences by capturing their attention and stimulating perception, cognition, and emotion. The grasp on the audience’s attention and generating certain kinds of emotions are driven by the structure of audio–video placement in a film. A neurocinematics study explores different brain processes and mental states while watching movies. In line with this, neuroaesthetic is the field that involves the study of esthetic processing in the brain while watching a structured video pertaining to a set of emotions. Esthetic components of audio–video stimuli evoke various emotions in our daily lives.

**Fig. 1:**
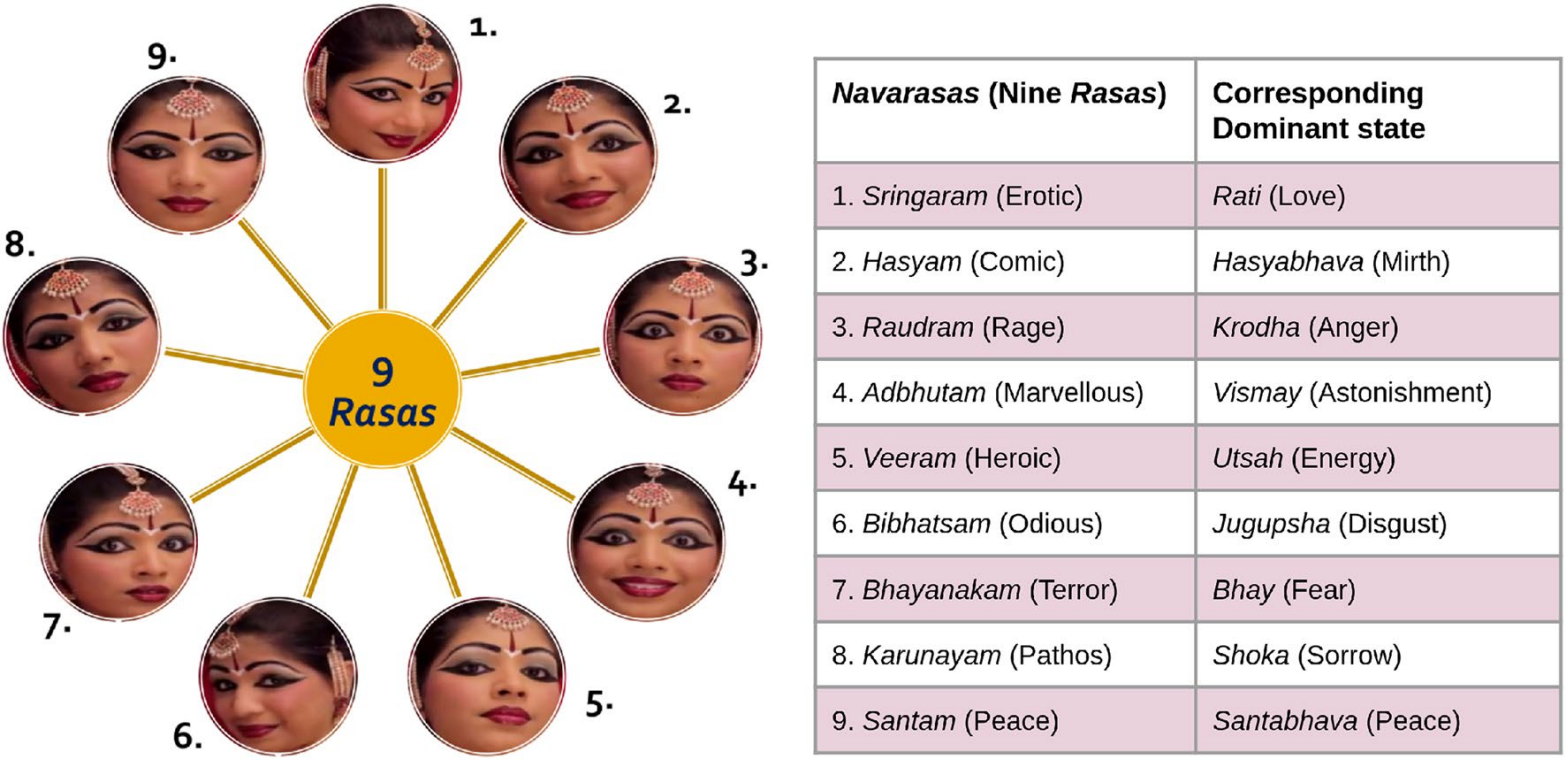
The nine-dimensional classification of emotions as described in $\textit{Natyashastra}$ (Indian $\textit{Rasa}$ Theory). The figure on the left depicts facial expressions corresponding to nine different $\textit{Rasa}$s. In the table we give closest English translation of these $\textit{Rasa}$s, and the corresponding dominant emotional state (or $Sthayi\ Bhava$) also with the meaning in English. (Image source: https://www.youtube.com/watch?v=sSdMUaF3-18)

The previous studies in neuroaesthetics are mostly based on the western classification of emotions. Several such categorizations of emotions have been discussed in the literature. Ekman discusses the six basic emotions including anger, disgust, fear, joy, sadness, and surprise [1], besides another six categorizations comprising desire, love, sorrow, wonder, happiness, and interest [2, 3]. Tomkins et al. [4] in their approach to emotion, describe nine basic emotions including anger, contempt, disgust, distress, fear, interest, joy, shame, and surprise. The cognitive structure of emotions has also been discussed in further 22 forms. In this study, we present our work on the Indian categorization of emotions into nine classes as described in ‘Natyashastra’: a treatise on performing arts. These nine dimensions of emotions correspond to the nine $\textit{Rasa}$s (esthetic impact of an artwork). We study these $\textit{Rasa}$s as evoked via watching audio-visual entertainment (movie clips) through electroencephalographic recordings. A $\textit{Rasa}$ describes a state of mind to indicate emotion. This research is pivotal in understanding the theoretical work done by researchers in the domain of neuroaesthetics, especially Indian esthetics of performing arts. Several works on $\textit{Rasa}$s have been produced, including dance, drama, and paintings [5–7]. However, there is a need to understand the underlying cognitive processes while observing various $\textit{Rasa}$ forms. This article investigates the role of different brain oscillations while watching nine $\textit{Rasa}$s in the form of audio-visual clips.

**Fig. 2:**
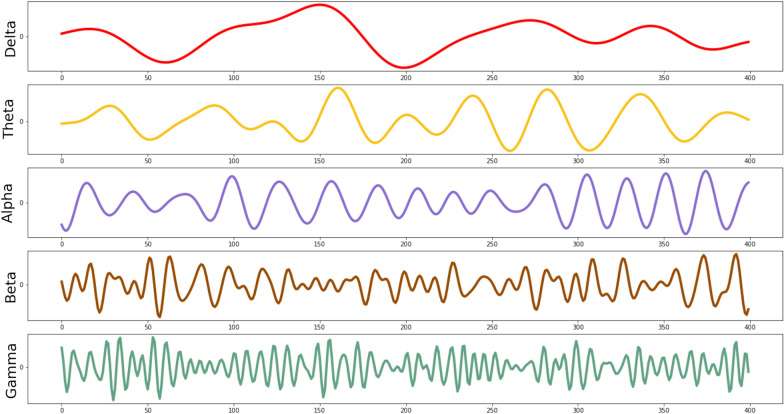
Brain rhythms depict the primary five waveforms. The figure shows various frequencies present in the $\text {EEG}$ signal. Delta band (1–4 Hz) depicts lowest frequency waves, followed by theta band (4–7Hz), alpha band (8–13 Hz), beta band (13–30 Hz) and gamma band (30–45 Hz)

This study recorded electroencephalography (EEG) responses while participants were watching movie clips depicting nine $\textit{Rasa}$s. EEG has been a principal tool for brain research because it reflects electrophysiological activity that is representative of brain function and EEG recording can be conducted at a relatively low cost with high temporal and useful spatial resolution [8]. EEG signals produce high-resolution images of neural oscillations, which opens several ways to study the human brain, from treating mental disorders to understanding emotions. For example, to study the brain processes involved during happy or sad emotions, participants can view emotional images while recording their brain activity [9]. This opens the research avenue to explore different emotional processes based on the stimuli. EEG signals represent synchronized electrical pulses from masses of neurons interacting with one other. Brain rhythms are primarily divided into five frequency bands, differentiated via their morphological and functional aspects. These are majorly classified into five frequency bands: delta (1– 4 Hz), theta (4– 7 Hz), alpha (8–13 Hz), beta (13–30 Hz), and gamma (30–45 Hz). Figure 2 displays the five brain rhythms.

Brain waves are the windows to understanding cognitive functions and their underlying brain processes. The morphology of EEG signals encodes complex properties and patterns, which can be decoded to connect previous knowledge to new ones to get more depth of brain processes. Slow-to-fast brain rhythms have been discussed widely spanning numerous domains of cognitive neuroscience. Delta rhythm is the slowest and strongest brainwave and is usually associated with the deepest form of dreamless sleep. Theta waves are observed in deep meditation and relaxation. Alpha band is associated with relaxation or calmness, and alertness. Beta band is marked by the state of wakefulness/consciousness, observed when performing any cognitive tasks (e.g., problem-solving, decision-making, etc.). Gamma frequencies are the fastest brainwaves, correlated with long-range neuronal communication, and facilitating the neural mechanisms underlying attention [10–13]. Previous studies on emotions highlight the discussion on frequency bands. Gamma band is ultra-fast brain waves identified to play an important role in human emotions [9, 14]. Gamma band is shown to find the differences between happy and sad emotions [15]. Beta bands are indicated for identifying three emotions: positive, neutral, and negative [16, 17]. A recent study finds the significance of beta and gamma bands in the discrimination of low/high valence, low/high arousal [18]. A study of event-related oscillations involving event-related synchronization/desynchronization has discussed the role of the slow (delta) waves in emotional processing in the passive viewing of emotionally evocative pictures [19].

Previous studies have suggested that functional connectivity in different frequency bands preserves significant network topology, which may be employed to classify emotions. In recent years, complex network theory has gained popularity [20], researchers have shown that EEG can be used to build brain networks and the resulting networks show a number of important topological traits [21]. A functional connection in the brain is defined typically as the temporal correlation between remote neurophysiological events [22]. Brain activities require interactions among multiple brain regions. Emotional processing requires the cooperation of many brain regions, as it is a high-level cognitive function [23]. The study of brain activity mechanisms often relies on brain networks, which depict relationships between brain regions and information exchange between them [24, 25]. Using functional connectivity, Zhang and colleagues identify the interaction of the prefrontal area to most other areas in emotional processing [26]. Gamma waves form more dense connections during the negative and neutral valences than beta waves with specific sites of right frontal and parietal–occipital regions [27]. According to previous research, functional connectivity measurements based on EEG data effectively generate the representation that may depict neural signatures for different emotional states.

Furthermore, functional connectivity has been studied widely by various graph theoretical measures, which reveal crucial topological features of the brain network [28]. Graph theoretical analysis of human brain networks has been utilized in a variety of imaging modalities, including EEG/MEG, functional MRI, diffusion MRI, and structural MRI [29]. The impact of emotional stimuli on large-scale functional brain networks can be measured through the evaluation of parameters such as centrality and global efficiency [9, 30]. Other network properties such as modularity, node betweenness centrality, clustering coefficient, and the existence of highly connected hub regions have been consistently discussed in the EEG studies [21, 29, 31]. Several network measures are explored to identify the characteristics of emotional states. Alpha frequency has been found to have the closest community structure across nine emotions [32]. Another study discussed that the clustering coefficient is higher in the left anterior regions of the negative emotions than positive groups [33].

Evidence from previous studies strongly suggests that functional connectivity of different frequency bands preserves significant network topology, which may be employed to classify emotions. In line with these findings, we extract network features from EEG responses for classification between $\textit{Rasa}$s. This research is motivated by the hypothesis that each $\textit{Rasa}$ may exhibit characteristics that are indistinguishable or distinguishable from one another. The following three points state the two primary objectives of this research and the expected outcome:

1. Which frequency band represents the maximum indistinguishable and distinguishable pair of $\textit{Rasa}$s?
2. What pair of $\textit{Rasa}$s are indistinguishable and distinguishable?
3. We anticipate that the results of our research will be in line with previous neuroimaging studies on emotion, especially on the role of fast brain waves in classifying emotions. Some of the indistinguishable and distinguishable pairs reflect the relationship on the pre-defined emotion model.

This work provides neural correlates of $\textit{Rasa}$s in the form of brain networks and identifies brain waves that distinguish them the most and the least. Our research provides insights into the brain processing of emotionally laden movie clips that elicit a certain mood. We believe that our analysis and results may provide opportunities for performers to understand the brain frequencies generated while doing an act among an audience; and the same goes for other art forms like music, literature and paintings. This is analogous to neural entrainment [34]—where a rhythmic sensory stimulus synchronizes neuronal activity. In the case of performing arts, the performer generates certain kinds of emotions that may induce the entrainment between the performer and viewer. Therefore, this research has potential implications for studying the entrainment of brain oscillations between performer and viewer. Such synchrony of oscillations are the key to generating better performances and a better viewer experience.

To the best of our knowledge, this is one of the first attempts at the scientific study of $\textit{Rasa}$s that involves modern experimental techniques and methodology, e.g., brain imaging through EEG, network construction based on weighted phase lag index, and machine learning for classification of $\textit{Rasa}$s. Such a study is novel and interesting, especially in the domain of neuroaesthetics, because $\textit{Rasa}$s are defined as the esthetics associated with an art form experienced by an audience, and are not pure emotional states. Through our analyses, we not only find differences and commonalities in how the nine $\textit{Rasa}$s are exhibited as brain waves, but also discover results that complement our contemporary understanding of emotions and brain waves.

This article is organized in seven sections: (a) Introduction, (b) The $\textit{Natyashastra}$ and $\textit{Rasa}$s, (c) Data description and preprocessing, (d) Methodology, (e) Results, (f) Discussion, (g) Limitations and future scope, and (h) Conclusion.

## The $\textit{Natyashastra}$ and $\textit{Rasa}$s

The ‘Natyashastra’ (NS), the ancient Indian treatise on performing arts, which dates back to the second century AD, provides a major basis for the Indian system of categorizing emotional states [35].

Attributed to Bharata Muni (Sage), the NS provides instructions on topics such as dramatic composition, structuring of a play, construction of the stage, acting styles, kinds of body movements, costumes, goals of the art director, etc. [36]. NS has not only influenced various literary traditions in India, such as dance, music, and acting but propounded $\textit{Rasa}$ Theory. The prime highlight of the theory is that although entertainment is the definite desired effect of performance art, it is not the primary goal. As a method of performance by movie actors, $\textit{Rasa}$, has been an undeniable part of Indian cinema (Bollywood). In contrast to western method acting, where an actor embodies the character they play, the focus of the $\textit{Rasa}$ method is to convey the emotion. Hence, according to $\textit{Rasa}$ theory, the performers must become the living embodiment of the $\textit{Rasa}$ they depict [37].

A word non-existent in the English language, $\textit{Rasa}$ expresses a combination of the ‘artist’ and the ‘aesthetic’ [38]. Its origins refer to the concept of taste of cuisine and can mean the essence or flavor. Bharata Muni described $\textit{Rasa}$ as ’extract’, to imply something worthy of being tasted, and asserted that without $\textit{Rasa}$ the purpose of art is unfulfilled [38]. In [39], $\textit{Rasa}$ is described as an ‘ecstasy’ caused by watching or listening to an art form such as a play or music. Additionally, as opposed to being a single pure thing, $\textit{Rasa}$ is a superposition of many sensory inputs that produce “a richly textured, emotionally resonant experience larger than the sum of its parts” [40]. These parts (or ingredients, described in analogy to a cuisine) of $\textit{Rasa}$s are the bhavas. These distinguishable bhavas (emotional states), when combined creatively, add to give enjoyable esthetics of a mixture of emotions. Bharata describes $\textit{Rasa}$s as “moods” experienced by the audience, and bhavas are “state of being” portrayed by actors in performing arts. He describes $\textit{Rasa}$s and bhavas as “cause one another to originate”. Uppal (2018) [38] describes $\textit{Rasa}$s as taste in food, or melody in music, or movement of the body in a dance, while the bhavas are more discretely conveyed through words, gestures, acting, expressions, etc. In light of this definition of the $\textit{Rasa}$, and the traditional pertinence of $\textit{Rasa}$ theory in Indian cinema, we design our study and look at it through the lens of modern theories of cognition, perception, and computational esthetics.

In the Natyashastra, *Rasa*s (pg. LXXXVI: [41]) are considered as superposition of certain dominant states (sthayi bhava), transitory states (vyabhicari bhava), and temperamental states (sattvika bhava) of emotions (pgs. 102, 105: [41]). Out of these only the the sthayi bhava is transformed into $\textit{Rasa}$ [38]. There are eight $\textit{Rasa}$s in classical Indian performing arts which are: Sringaram (erotic), Hasyam (comic), Karunayam (pathetic), Raudram (furious), Veeram (heroic), Bhayanakam (terrible), Bibhatsam (odious), and Adbhutam (marvelous). A later addition to the Sanskrit poetic tradition is a ninth sentiment called Santam (peace) (pg. 102: [41]). The facial expressions and the dominant state (bhava) corresponding to each of these $\textit{Rasa}$s are depicted in Fig. 1 We based our selection of movie clips on this classification system and chose ones that correspond to each $\textit{Rasa}$. In light of the fact that there are no defined movie clips for this classification system, the movies we selected represent one set of selections.

## Data description

The Institute Ethical Committee (IEC) of Indian Institute of Technology, Gandhinagar, approved this study. Prior to conducting experiments, all of the participants provided informed consent.

### Subjects

The study involved 20 healthy (mean age: 26 years, 16 males, 4 females), right-handed students from Indian Institute of Technology Gandhinagar. All participants were proficient in the Hindi language, which was also the language of the video clips. All participants were briefed about the task and asked to maintain their attention while watching the film clips. Small groups of subjects independently scored movie clips from each category of emotion. Only those clips were selected with the highest ranking for evoking a particular response for all categories.

**Table 1:** Movie clips used in EEG data collection

Movie ID	Rasa genre	Film name	Director	Year	Duration	Start time	End time
1	Adbhutam	Mr. India	Shekhar Kapur	1987	1m 48s	1h 1m 40s	1h 3m 28s
2	Bhayanakam	Bhoot	Ram Gopal Varma	2003	1m 34s	1h 2m 57s	1h 4m 31s
3	Bibhatsam	Rakhta Charitra	Ram Gopal Varma	2010	1m 12s	43m 55s	45m 7s
4	Hasyam	3 Idiots	Rajkumar Hirani	2009	2m 33s	59m 55s	1h 2m 28s
5	Karunayam	Kal Ho Naa Ho	Nikhil Advani	2003	2m 37s	2h 47m 41s	2h 50m 18s
6	Raudram	Ghajini	A.R. Murugadoss	2008	2m 9s	2h 38m 43s	2h 40m 52s
7	Santam	Zindagi Na Milegi Dobara	Zoya Akhtar	2011	2m 22s	48m 22s	50m 44s
8	Sringaram	Umrao Jaan	Muzaffar Ali	1981	42s	43m 08s	43m 50s
9	Veeram	Lagaan: Once Upon a Time in India	Ashutosh	2001	2m 3s	2h 10m 57s	2h 13m

### Audio-visual stimuli

Bollywood is popular Indian cinema based on the Hindi language. We selected nine Bollywood movie clips covering four decades from the 1980s to recent, as shown in Table 1. These movie clips depicted each $\textit{Rasa}$ and selection was based on the independent rating from a small group of participants. Each film segment had a different length because the clips contained narration that had to be shown for a certain time to evoke a specific $\textit{Rasa}$. Film clips ranged in length from 42 s to 2 min 37 s, as shown in Table 1.

### EEG data acquisition and preprocessing

EEG recordings were collected while a participant was asked to watch the selected nine film clips corresponding to nine $\textit{Rasa}$s. A high-density Geodesic system of 128 channels was used for this acquisition with a sampling rate of 250 Hz. A white fixation cross on a blank screen preceded each film clip for 10 s, and the order of the films was randomized for each participant. The complete experiment was designed and run in E-primeTM and recordings were captured using Net-stationTM. The preprocessing was performed using the Matlab EEGLAB package. High-frequency signals after 60 Hz were filtered to avoid noise effects. Raw EEG data mostly contain movement and eye blink artifacts that can be checked carefully and removed to make data useful for analysis. Therefore, we applied artifact subspace reconstruction to keep the clean continuous data [42]. Following this, we chunked the data respective to each $\textit{Rasa}$ across subjects and used it for further analysis.

**Fig. 3:**
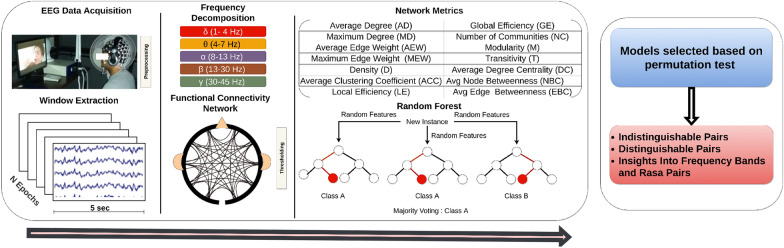
The workflow of the present paper: (Box 1) EEG data acquisition is performed when a participant watches movie clips. The subsequent step involves preprocessing and segmentation of EEG signals into epochs of 5 s. Then the extracted segments are passed for frequency decomposition into five frequency bands comprising delta (1–4 Hz), theta (4–7 Hz), alpha (8–13 Hz), beta (13–30 Hz), and gamma (30–35 Hz) bands. This is followed by the construction of brain networks with a threshold that retains the significant connections. Network properties are computed from the thresholded functional networks. These network properties are then used as features to build binary classifiers between $\textit{Rasa}$s. Resultant models are selected based on the significance of the permutation test (Box 2). From the selected models, we identify the distinguishable and indistinguishable pairs and the frequency bands in which these pairs appear

## Methodology

### Construction of brain networks

We constructed the functional connectivity networks using the EEG signals from each of the participants and for each of the $\textit{Rasa}$s. The nodes of these networks were the EEG electrodes and the edges representing the strength of connections between the nodes were evaluated using a measure called weighted Phase Lag Index (wPLI). The wPLI that quantifies the phase synchrony between any two time-varying signals, is a standard functional connectivity measure used in the network neuroscientific community. The wPLI is defined as the extent of absolute phase lag or lead between two signals weighted by the imaginary component of the cross-spectral power density between these signals. It is robust to the volume conduction, presence of noise, and biases induced by sample size in the electrophysiological data [43–45]. Firstly, the EEG time-series signals from each of the electrodes were segmented into 5-s-long windows or epochs with an overlapping window of length 2.5 s. Followed by filtration in five frequency bands, namely, delta: 1–4 Hz, theta: 4–7 Hz, alpha: 8–13 Hz, beta: 13–30 Hz, and gamma: 30–45 Hz. The wPLI measure between a pair of signals calculates the average over the number of epochs. For our computation of wPLI, we used MNE-python’s connectivity module [46]. This gives us the five different coupling matrices (weighted adjacency matrix) each of size (128$\times$128) pertaining to each of the five frequency bands, for each participant and each $\textit{Rasa}$.

### Thresholding of brain networks

Functional networks mostly preserve weak and erroneous connections, which may conceal the topology of crucial connections [21]. Thresholding is commonly used to remove a percentage of the weakest links to retain a usable sparse network. We applied the thresholding process as implemented in the paper [47]: the network should be 97% connected, and the average degree should be greater than $2*log(n)$, while maintaining the highest threshold value for edge weights, where n is the number of nodes.

### On the choice of network metrics as features

We chose 14 structural metrics calculated from the final weighted and thresholded brain networks as features. These were: average degree, maximum degree, average edge weight, maximum edge weight, network density, average clustering coefficient, local efficiency, global efficiency, number of communities, modularity, transitivity, mean degree centrality, mean node betweenness centrality, and mean edge betweenness centrality. As stated before, we hypothesize that the network measures obtained from the connection topology carry information specific to different $\textit{Rasa}$s in different brain frequency bands. This assumption is based on two of the research findings in neuroscience: one linking graph theory with brain conditions/states, and the other highlighting the role of frequency bands in brain processes. Several studies that use the graph theoretic framework [48, 49] to study the complex system of the brain, have proven that different structural and functional aspects of the brain are captured by EEG-based connectivity patterns of the brain network [30, 50–54]. These studies have highlighted that such brain functional networks can be characterized in terms of complex network properties, such as node betweenness, small-worldness, hubs, and modularity. Moreover, they demonstrated that these structural connectivity metrics could also distinguish between different cognitive states and pathophysiological states of brain [54]. Since our network connections are governed by the phase relationship of EEG signals between electrodes, they capture the functional dynamic connectivity pertaining to the activation of brain pathways of emotions. The brain frequencies as observed in clinical EEG, on the other hand, have played an enormous role in cognitive research [55]. Different frequency bands, their power content, and amplitude have been found to be specific to various basic cognitive engagement states such as wakefulness, sleep, and attention, brain diseases such as depression in Parkinson’s [56], and schizophrenia [57], the accuracy of working memory in adults [58] and encoding personality traits [59].

#### Definitions of network metrics

In this section, we define each of the network metrics [28] used as features in this work. We use the NetworkX Python library [60] to evaluate each of these metrics:

1. *Average degree (AD)*: The degree of a node in a network is the number of its neighbors or the number of nodes that it directly connects to. The average of this number over all the nodes in the average degree.
2. *Maximum degree (MD)*: It is the maximum of all the node degrees in a network.
3. *Average edge weight (AEW)*: Edge weight is the strength of an edge connecting given two nodes in a network. Average edge weight is the mean of edge weights over all the edges in the network.
4. *Maximum edge weight (MEW)*: It is the maximum of all the edge weights in the network. In other words, it is the strongest connection present in the network.
5. *Density (D)*: It is the ratio of the total number of edges present in the network to the number of possible edges in the network.
6. *Average clustering coefficient (ACC)*: The clustering coefficient of a node measures the fraction of triangles involving that node. In other words, it measures the extent to which its neighbors tend to form a complete graph. The average clustering coefficient is the average of this quantity over all nodes.
7. *Local efficiency (LE)*: For a network node, it is defined as the inverse of the average shortest path length of all its neighbors among themselves. It measures how robust the network is to the failure of this particular node in terms of its communication efficiency.
8. *Global efficiency (GE)*: Similarly, global efficiency measures the efficacy of distant information transfer in a network. It is defined as the inverse of the average characteristic path length between all node pairs present in the network.
9. *Number of communities (NC)*: A community in the network is the set of nodes that have denser connections or a higher number of edges within this node-set, than to other nodes or communities in the network. A modular network is organized into clearly identifiable communities.
10. *Modularity (M)*: Modularity is the measure of the extent to which a network is divided into communities. This measure is often used as a quantity that is optimized, in various community detection algorithms.
11. *Transitivity (T)*: Transitivity is the ratio of thrice the number triangles to the number of connected triples of nodes in the network.
12. *Average degree centrality (ADC)*: Centrality is the measure of the importance of the node in a network, or how central is the node to overall network connectivity. The degree centrality of a node is a fraction of the number of links a node has to the total number of potential links it can have in the network.
13. *Average node betweenness centrality (NBC)*: Betweenness centrality measures how often a node bridges the connections between any two pairs of nodes in a network via the shortest path. If a node lies in a large number of such shortest paths, it has a high node betweenness centrality. Average NBC is the average over all nodes.
14. *Average edge betweenness centrality (EBC)*: Similarly, for an edge, the edge betweenness centrality measures the number of shortest paths on the network to which this edge belongs. Average EBC is average over all network edges.

### Random Forest (RF) classifiers

Network metrics from different networks were used as features for the classification. In this study, we trained binary classifiers between two given $\textit{Rasa}$s. We selected Random Forest (RF) classifier for this research due to its well-established theory and easy interpretability [61]. RF predicts the class based on a number of fitted decision tree classifiers on various sub-samples of the dataset. Features are used to build decision trees, where a feature denotes a node, and a threshold is used to split the node into two children nodes. The quality of the split is decided using the Gini criteria. Once the trees are fitted, and optimum thresholds are identified, the final class is selected by the majority vote. RF controls over-fitting and averaging improves the predictive accuracy. We performed validation using the tenfold stratified technique. The classifier’s performance was evaluated using accuracy, precision, recall, and f1-score. Models were developed using scikit-learn python [64]. The complete process of construction of networks to classification is shown in Fig. 3. The input to the random forest was the number of subject samples $\times$ 14 features. The ‘number of the tree’ was set to 100 trees in the forest, the ‘quality of the split’ was measured by Gini impurity, ‘max depth’ of the nodes of the trees were spread until all leaves were pure or leave had minimum split samples. The ‘min samples’ were set to 2 for the minimum number of samples required to split an internal node. ‘Min sample leaf’ was set one for the minimum number of samples required to be at a leaf node. ‘Min weight fraction’ on the leaf was set to equal weight, and the ‘max features’to consider when looking at the split was sqrt(number of features).

Previous studies have suggested employing permutation-based p-values for assessing the competence of a classifier [62, 63]. This test is proposed to measure the real connection between the data and the class labels, and learning signifies a real class structure. We used the permutation test with 10,000 rounds with fivefold cross-validation to examine the statistical significance of the classifier. The permutation test shuffles the labels of the instances to evaluate the significance of the classifier. This test [63] has been utilized extensively in the literature and the results discussed via the permutation test are effective. A small p-value suggests that there is a real dependency between features and targets, which has then been used by the estimator to give good predictions. A high *p*-value may indicate little or no relationship between the features and targets or that the estimator could not use the relationship to make good predictions. Majorly, the permutation test procedure assesses how likely a particular accuracy score would be observed by chance. We have used the implementation of sklearn [64].

### Visualization

The obtained feature matrix was high dimensional, which limits the visualization in two-dimensional space; therefore, we applied t-distributed Stochastic Neighbor Embedding (t-SNE) to generate lower-dimensional embedding [65]. t-SNE is a manifold learning unsupervised approach for non-linear dimensionality reduction. It transforms the data into a low-dimensional space for visualization. EEG signals contain non-linearity and represent manifold brain processes, therefore we applied this technique to observe the manifolds that can retain the non-linear relationship of the dataset. There is one parameter, perplexity, which defines the variance of the Gaussian distribution. Different values of perplexity result in significantly different results, hence, we generated two-dimensional embedding using seven values: [5, 10, 15, 20, 30, 40, 50].

### Statistical analysis

To test for gender and age effects on the features extracted, we averaged the network features extracted for male and female participants for each emotion and found that the measures for the two genders were strongly correlated ($R^{2}>0.95$). Similarly, we did not find any correlation between age and any of the features extracted ($p>0.05$). Hence, we do not consider these two factors in further analyses.

**Fig. 4:**
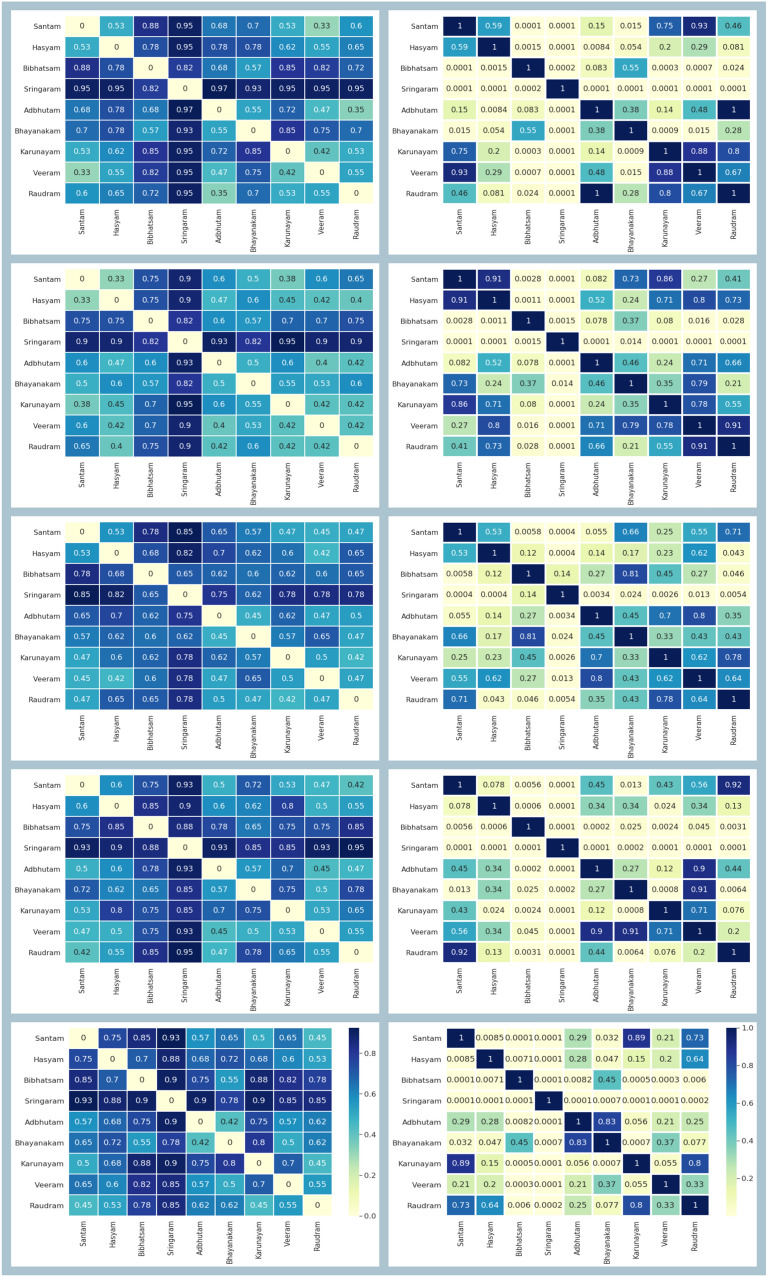
[Left column] Matrices represent the test accuracy between each pair of $\textit{Rasa}$s across five frequency bands. Order of frequency bands from top to bottom is: delta, theta, alpha, beta, and gamma. [Right column] The corresponding p-value indicates the statistical significance of test scores between $\textit{Rasa}$ pairs

## Results

### Findings from classification

We developed binary classification models between pairs of $\textit{Rasa}$s (emotions) across five bands. There were 36 models built for each band comprising a total of 180 (36 $\times$ 5) models. In Fig. 4, the first column depicts the test accuracy score for each model across bands and the second column mentions the respective significance scores (*p*-values). Based on the *p*-value, we segregated the $\textit{Rasa}$ pairs as either indistinguishable or distinguishable. Indistinguishable refers to the pair whose *p*-value was greater than 0.1, whereas the distinguishable pair had p-value less than 0.01.

#### Definition

Indistinguishable pair implies that the classification model was unable to discriminate between features of $\textit{Rasa}$s. In contrast, distinguishable pair represents that the model determined the discriminating properties between $\textit{Rasa}$s.

**Fig. 5:**
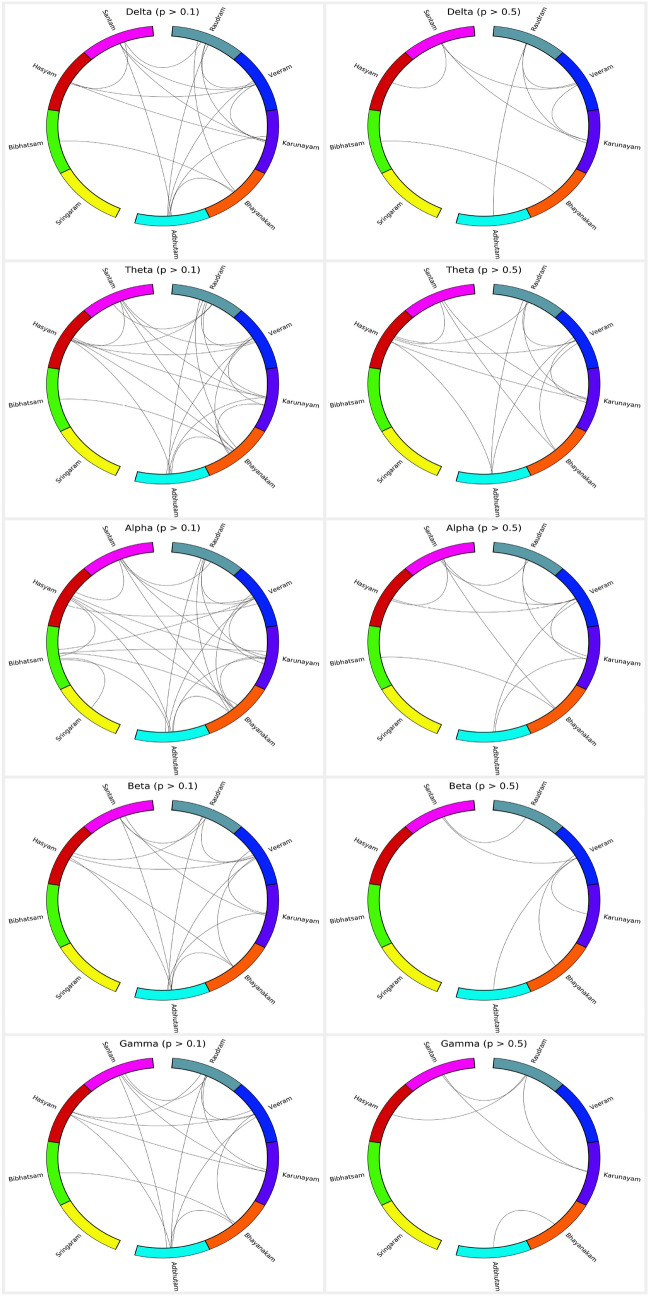
A connection between two $\textit{Rasa}$s represents an indistinguishable pair. The top and bottom rows represent the connections obtained with a *p*-value greater than 0.1 and 0.5, respectively. Indistinguishable pair implies that the model was unable to distinguish between characteristics of $\textit{Rasa}$s. [From the top, in the anticlockwise direction the $\textit{Rasa}$s are in order: Santam (pink), Hasyam (red), Bibhatsam (green), Sringaram (yellow), Adbhutam (cyan), Bhayanakam (orange), Karunayam (purple), Veeram (blue), and Raudram (dark green).]

**Fig. 6:**
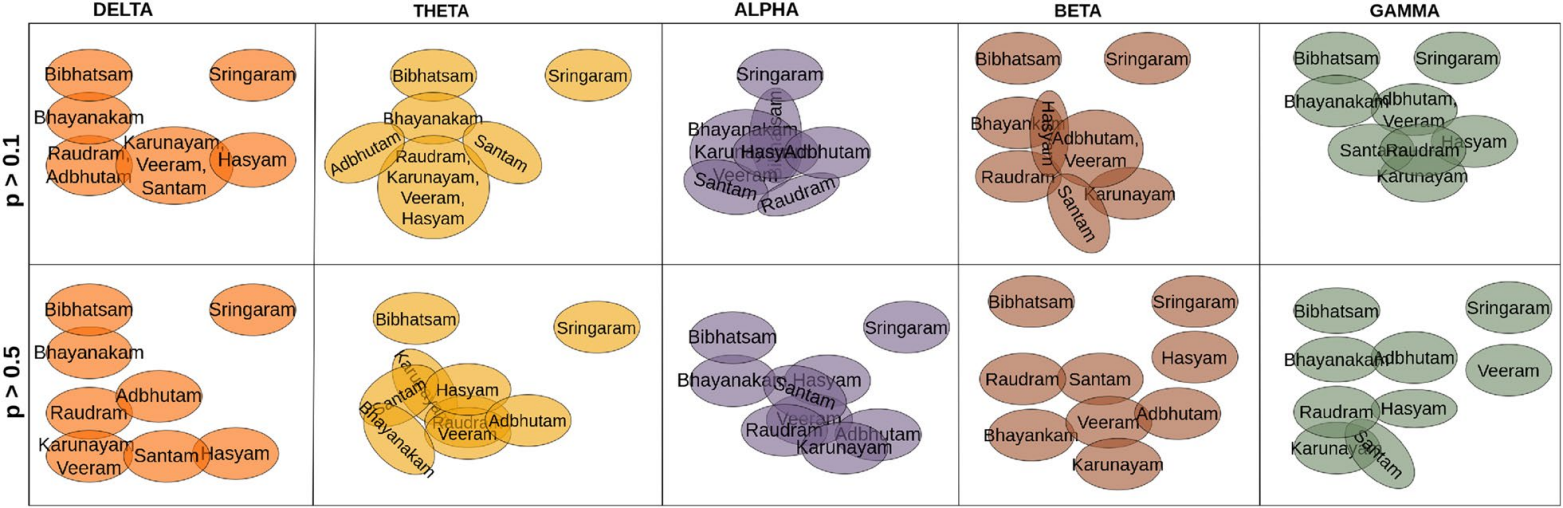
The top and bottom rows represent the Venn diagrams obtained with a *p*-value greater than 0.1 and 0.5, respectively. The presence of more than one $\textit{Rasa}$ in a set indicates similar indistinguishable connections

#### Indistinguishable pairs

We selected the indistinguishable pairs based on the two thresholds on the *p*-value, i.e., $p > [0.1, 0.5]$, and plotted them as shown in Fig. 5. The nine $\textit{Rasa}$s are arranged in a circular layout and the existing links between the $\textit{Rasa}$ pairs represent that they are indistinguishable in a given band. The maximum number of such pairs were found in the alpha and theta bands; whereas, the delta, beta, and gamma bands showed lesser pairs. To illustrate the indistinguishable pairs more clearly, we constructed Venn diagrams based on the obtained relationships, as shown in Fig. 6. The overlap between any two $\textit{Rasa}$s depicts that the pair is indistinguishable. One such example is that of Bibhatsam and Bhayanakam, a pair that is largely indistinguishable, except in the beta band. However, for $p > 0.5$ they formed a indistinguishable pair only in delta and alpha bands.

*Key finding:* Theta and alpha bands formed maximum indistinguishable pairs.

**Fig. 7:**
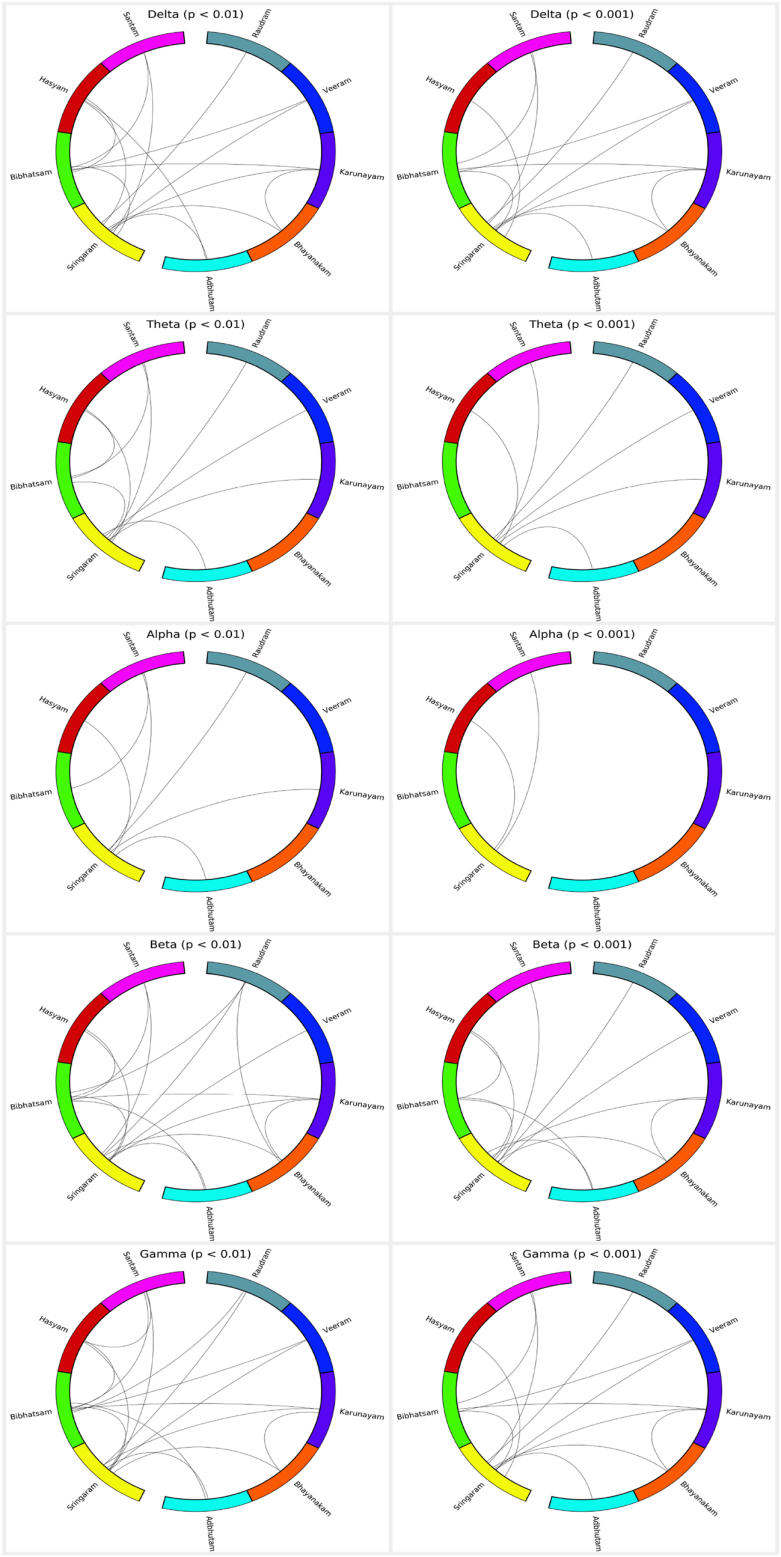
A connection between two $\textit{Rasa}$s represents a distinguishable pair. The top and bottom rows represent the connections obtained with a *p*-value less than 0.01 and 0.001, respectively. Distinguishable pair implies that the model was able to distinguish between characteristics of $\textit{Rasa}$s. [From the top, in the anticlockwise direction the $\textit{Rasa}$s are in order: Santam (pink), Hasyam (red), Bibhatsam (green), Sringaram (yellow), Adbhutam (cyan), Bhayanakam (orange), Karunayam (purple), Veeram (blue), and Raudram (dark green).]

**Fig. 8:**
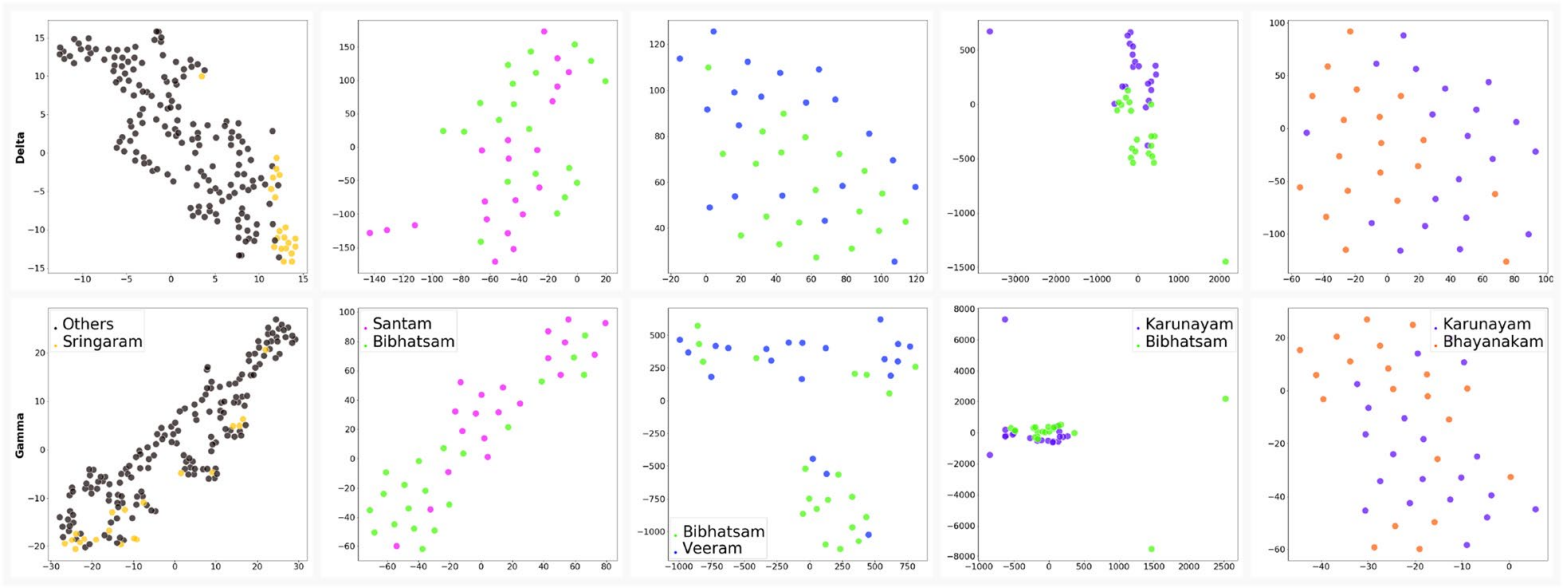
Network features of a distinguishable pair ($p < 0.001$) are projected in lower-dimensional 2D space using t-SNE. The top and bottom rows represent the features extracted from delta and gamma bands, respectively

**Table 2:** The classifier’s performance is evaluated using accuracy, precision, recall, and F1-Score

Class1	Class2	Band	Accuracy	Precision	Recall	F1-Score	*p*-value
Bibhatsam	Santam	Delta	0.875	0.9	0.85	0.8666	0.00009
Bibhatsam	Veeram	Delta	0.825	0.8833	0.8	0.81	0.0006
Bibhatsam	Karunayam	Delta	0.85	0.8833	0.85	0.8433	0.0002
Bhayanakam	Karunayam	Delta	0.85	0.8833	0.85	0.8433	0.0009
Bibhatsam	Santam	Gamma	0.85	0.9	0.84	0.84	0.00009
Bibhatsam	Veeram	Gamma	0.825	0.8833	0.8	0.81	0.0002
Bibhatsam	Karunayam	Gamma	0.875	0.9666	0.8	0.8466	0.0004
Bhayanakam	Karunayam	Gamma	0.8	0.8	0.9	0.8233	0.0006

*p*-value is obtained by permutation test

#### Distinguishable pairs

The smaller the *p*-value, the stronger the evidence to have the discriminating features between two classes. Therefore, we selected two thresholds ($p < [0.01, 0.001]$) and, respectively, plotted the distinguishable pairs in Fig. 7. The alpha band formed the minimum distinguishable pairs followed by the theta band, whereas the delta, beta, and gamma bands revealed the maximum distinguishable pairs. The delta and gamma bands showed a similar set of distinguishable pairs when $p < 0.001$. Sringaram reflected the significant distinction from other $\textit{Rasa}$s across bands, and in the delta band it showed a classification accuracy of above 90% ($p < 0.001$) with other $\textit{Rasa}$s except for Bibhatsam. Bibhatsam formed a discrimination group ($p < 0.001$) with Santam, Veeram, Karunayam, and Sringaram, with accuracies of 88%, 82%, 85%, and 82%, respectively. Theta band showed distinguishable pairs of Sringaram with six other $\textit{Rasa}$s with accuracy approximately above 90%, except for Bibhatsam and Bhayanakam. The alpha band for Sringaram formed only two discriminating pairs ($p < 0.001$) with Santam and Hasyam. For Sringaram, beta and gamma bands showed a similar relationship as depicted in the delta band. In the beta band, Bibhatsam formed two pairs ($p < 0.001$) with Hasyam and Adbhutam. Gamma band revealed the same pairs as delta. Bhayanakam with Karunayam depicted significant discrimination across the delta, beta, and gamma bands. In Table 2, the classifier’s performance is shown for delta and gamma bands.

We projected the feature matrix to a lower-dimensional space, which made it easier for interpretation. We applied an unsupervised t-SNE dimensionality reduction technique on the obtained distinguishable pairs ( $p < 0.001$) in the delta, and gamma bands. We observed clear separation in some pairs as shown in Fig. 8. For example, Sringaram’s data points clustered mostly in a corner of the 2-dimensional feature space separated from the other $\textit{Rasa}$s. Secondly, Karunayam with Bibhatsam and Bhayanakam reflected a clear separation of data points in delta and gamma bands. Similarly, Bibhatsam showed spatial separation with Santam and Veeram $\textit{Rasa}$s. We rendered the 2D view using t-SNE, but there might be better separability in the higher dimensions.

*Key finding:* Slow wave (delta band) and fast wave (beta and gamma bands) formed maximum distinguishable pairs.

**Fig. 9:**
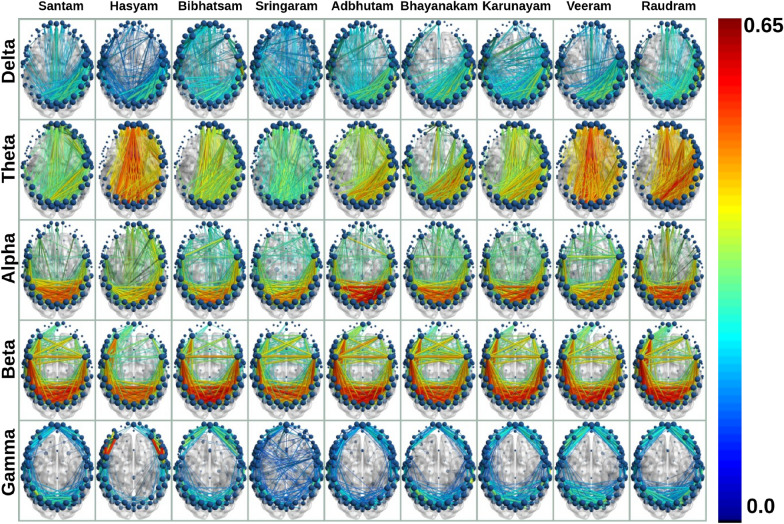
Connectivity graphs of $\textit{Rasa}$s depict 5% of the strong connections across bands. The node’s size indicates the degree, and the width and color of the edges denote the connection strength measured using wPLI index (averaged over all the 20 participants). Blue and red colors indicate the minimum and maximum strength, respectively. The visualizations are generated using the ’BrainNet Viewer’ (www.nitrc.org/projects/bnv/)

### Interpreting outcome of classifiers using network metrics

In this section, we aim to obtain an intuitive understanding of the classification results obtained in the previous sub-sections by analyzing the network properties of the different brain networks. In this pursuit, we take two approaches, one where the network metrics are averaged across $\textit{Rasa}$s for each frequency bands, and second, where the network metrics are averaged across frequency bands for each $\textit{Rasa}$.

#### Analyzing frequency bands after averaging network metrics across $\textit{Rasa}$s

For each frequency band, we averaged the magnitude of network metrics over all $\textit{Rasa}$s. The averaged metrics are shown in Table 3, with maximum and minimum values across the bands shown in bold fonts. From these values, we examine the similarities and differences between bands. Gamma band showed the minimum average degree, followed by the delta and beta bands. The maximum degree is observed in theta and alpha bands. Gamma had the minimum average edge weight, whereas alpha had the maximum value. The network density was minimum in the gamma band, followed by delta, beta, alpha, and theta bands. Delta band had the minimum average clustering coefficient, whereas the maximum was in the alpha band. Similar observations were repeated for the rest of the network metrics. Delta or gamma band indicated the minimum magnitudes of network metrics, whereas alpha or theta band maintained the maximum value. There were only two exceptions where the role was reversed—gamma band showed a maximum, and theta band a minimum. In contrast, average node and edge betweenness centrality (NBC, EBC) showed a descending order of magnitudes from gamma, delta, beta, alpha, and theta bands. We drew the top 5% of the network connections in Fig. 9.

*Key finding:* Delta and gamma bands have lower magnitudes of network metrics, whereas theta and alpha bands retained higher magnitudes, except for NBC and EBC.

**Table 3:** Frequency bands after averaging across all $\textit{Rasa}$s

Band	AD	MD	AEW	MEW	D	ACC	GE	LE	NC	M	T	ADC	NBC	EBC
Delta	19.1262	63.4888	0.4605	**0.7311**	0.1506	**0.2593**	0.5050	**0.4550**	4.2611	**0.0110**	0.2434	0.1506	0.0087	0.0019
Theta	**29.5588**	75.3388	0.5033	0.7966	**0.2327**	0.3262	**0.5625**	0.5495	4.2444	0.0119	0.3319	**0.2327**	**0.0071**	**0.0012**
Alpha	26.1502	**75.8944**	**0.5395**	0.8440	0.2059	**0.3955**	0.5444	**0.5956**	**4.2777**	**0.0270**	**0.3609**	0.2059	0.0077	0.0014
Beta	20.9482	70.3	0.4262	0.7958	0.1649	0.3873	0.5137	0.5708	4.2388	0.0191	0.3453	0.1649	0.0086	0.0019
Gamma	**12.6756**	**62.3611**	**0.416**	**0.8448**	0.0998	0.3253	**0.4661**	0.4872	**3.9333**	0.0239	**0.2392**	**0.0998**	**0.0101**	**0.0030**

Maximum and minimum values are highlighted

**Table 4:** Average network metrics for each $\textit{Rasa}$ obtained after averaging across all bands

$\textit{Rasa}$	AD	MD	AEW	MEW	D	ACC	GE	LE	NC	M	T	ADC	NBC	EBC
Raudram	**24.0454**	**74.76**	0.4527	0.8040	**0.1893**	**0.3630**	**0.5334**	**0.5653**	4.18	0.0099	**0.3235**	**0.1893**	**0.0079**	**0.0016**
Santam	23.2392	73.4	0.4291	0.7757	0.1829	0.3586	0.5298	0.5589	4.08	0.0108	0.3217	0.1829	0.0081	0.0017
Karunayam	23.0126	73.83	**0.4245**	**0.7712**	0.1812	0.3601	0.5290	0.5564	**3.97**	**0.0070**	0.3201	0.1812	0.0082	0.0017
Hasyam	22.7248	70.01	0.4425	0.7894	0.1789	0.3548	0.5241	0.5534	4.12	0.0177	0.3136	0.1789	0.0083	0.0018
Adbhutam	22.6998	71.87	0.4608	0.8037	0.1787	0.3529	0.5239	0.5446	4.15	0.0089	0.3155	0.1787	0.0083	0.0018
Veeram	22.6351	73.98	0.4492	0.8048	0.1782	0.3608	0.5263	0.5594	4.17	0.0136	0.3170	0.1782	0.0082	0.0018
Bhayanakam	20.6726	67.31	0.4749	0.8079	0.1627	0.3262	0.5108	0.5180	4.28	0.0146	0.3025	0.1627	0.0086	0.0019
Bibhatsam	19.9148	64.02	0.5010	0.8190	0.1568	0.3139	0.5052	0.5007	4.28	0.0215	0.2897	0.1568	0.0088	0.0020
Sringaram	**16.2820**	**56.11**	**0.5878**	**0.8463**	**0.1282**	**0.2582**	**0.4827**	**0.4274**	**4.49**	**0.0631**	**0.2336**	**0.1282**	**0.0096**	**0.0025**

Maximum and minimum values are highlighted

#### Analyzing $\textit{Rasa}$s after averaging network metrics across frequency bands

For each $\textit{Rasa}$, the magnitude of network metrics after averaging over the five frequency bands is shown in Table 4, with the minimum and maximum values highlighted in bold. The minimum and maximum average degrees were indicated by Sringaram (16.28) and Raudram (24.04). The maximum degree was found in three sets that had magnitude above 50, 60, and 70. Sringaram had the least maximum degree of 56.11, Bibhatsam (64.02) and Bhayanakam (67.31) formed another group of above 60. Hasyam (70.01), Adbhutam (71.87), Santam (73.4), Karunayam (73.83), Veeram (73.98), and Raudram (74.76) were above 70. The average edge weight between 0.40 and 0.46 comprised Karunayam (0.424), Santam (0.429), Hasyam (0.442), Veeram (0.449), and Raudram (0.45). Adbhutam (0.46), Bhayanakam (0.47), and Bibhatsam (0.50) observed within 0.52. And the maximum was for sringaram (0.58). Density ranges from 0.17 to 0.19 included Raudram (0.189), Santam (0.182), Karunayam (0.181), Hasyam (0.1789), Adbhutam (0.1787), and Veeram (0.1782). Bhayanakam (0.16), Bibhatsam (0.15), and Sringaram (0.12) had the least three values. For the remaining metrics (before ADC, as shown in Table 4), we found that Sringaram had minimum magnitude, whereas Raudram and Karunayam had maximum. In contrast, average node and edge betweenness centralities showed minimum values for Raudram and maximum for Sringaram.

**Fig. 10:**
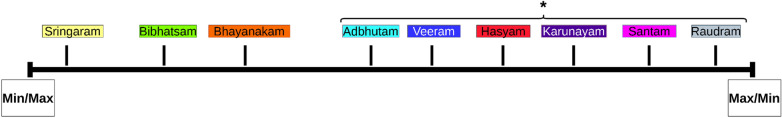
Network $\textit{Rasa}$ scale: Sringaram and Raudram form the limiting boundaries for the magnitude of network properties, and all the other $\textit{Rasa}$s fall within those limits. (*) over a set of $\textit{Rasa}$s denotes that their order is not necessarily the same as shown, and it may slightly vary with frequency bands. For some network metrics, they may share the properties or may differ. Bibhatsam and Bhayanakam are consistently close to each other across network metrics and also across the bands

*Key findings:*

- Ten out of fourteen network properties suggested that the $\textit{Rasa}$s Sringaram and Raudram limit the magnitude of network features. Based on this, we inferred a magnitude scale as shown in Fig. 10, where Sringaram determined the one side limit of the scale, while Raudram maintained the other side.
- In contrast to other network metrics, node, and edge betweenness centralities are maximum in the Sringaram and minimum in Raudram.
- The network properties of Bibhatsam and Bhayanakam were nearly close to each other.

## Discussion

Higher frequency has been consistently reported to be crucial for the classification of different emotions [66–68]. In the previous study for the classification of happiness and sadness, the gamma band has been the optimal band for generating discriminating features [15]. A recent study [33] presents that the beta and gamma are more effective brain rhythms in identifying emotions than the theta and alpha. Furthermore, some neuroscience studies reveal that neural encodings of emotional information are stored primarily in higher frequency bands [69, 70]. Another recent paper by Yang and colleagues reports that long-distance connections noted in the high-frequency bands, especially in the high gamma bands, showed significant differences among emotional states [9]. Brain activities in the high-frequency band (> 30Hz) are known to be associated with emotional integration and play a role in cognitive control of emotions [71, 72]. Several studies have looked at those high-frequency responses to affective pictures, most of which reported enhanced responses to negative images [73–75]. Zheng and colleagues observe that the delta band performed better than the theta and alpha bands for emotion recognition of three categories (positive, neutral, and negative) [76]. They observe this outcome from the features of differential asymmetry and rational asymmetry. Delta band is less studied in the literature, and a recent study on event-related emphasizes the research on delta activity patterns and alterations in delta energy, which might improve our understanding of emotional processing by focusing on the slow waves (delta band) [19].

Interestingly, our result on the alpha band resonated with the previous research on Indian $\textit{Rasa}$s [32]. This study reports that the community structure of different $\textit{Rasa}$ networks in the alpha band is the most similar. Similar observation about the indistinguishability of (two) emotions in alpha band was also reported in [77]—a study aimed at discriminating multiple emotional states using EEG data collected from subjects watching emotion-inducing video clips. According to another recent study, emotional stimulus processing is associated with a decrease of power in the alpha and beta bands across studies and task conditions [78].

**Fig. 11:**
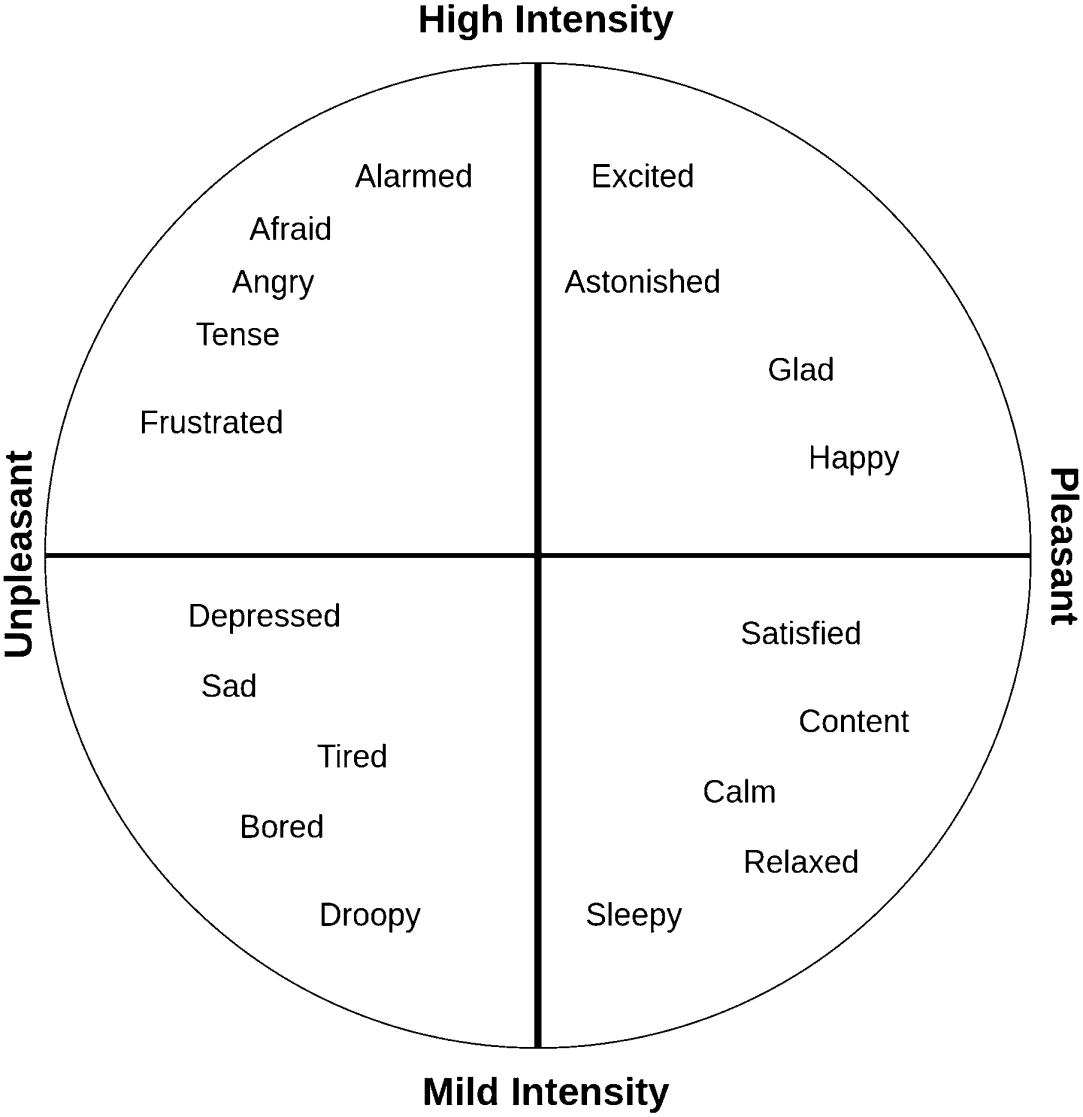
A circumplex model of emotions classification. The model has two dimensions encompassing pleasure and activeness

Most EEG studies include 15-20 participants because of the complexity in EEG setup and data collection. Therefore, with a small number of samples, some techniques are proposed to identify the significance of the Machine Learning performance estimates. The recent article [79] highlights the strong biases observed using solely K-fold cross-validation, and therefore it is significant to use rigorous methods for analysis. Hence, our study mainly used fivefold cross-validation with permutation test of 10,000 rounds that produced robust and unbiased performance estimates regardless of the sample size. Based on our results from classifiers and magnitudes of network metrics, we observe that Bhayanakam (fear) and Bibhatsam (disgust) exhibited high similarity. They both signify unpleasant emotions as per the circumplex model. Russell and James proposed a circumplex model for emotions classification [80]. This is more related to dimension space theory, which refers to emotion as continuous and relevant [81]. The circumplex model describes emotion into two dimensions: pleasure and activeness, as shown in Fig. 11. Activeness is categorized into mild and high intensity, while pleasure is classified into pleasant and unpleasant. Based on the results of previous research conducted in various countries and regions, this model is mostly accurate and consistent [82, 83].

A summary of our results obtained on distinguishable pairs in different frequency bands is presented in Table 5. We observe that Bibhatsam (disgust), an unpleasant emotion, was distinguishable from Santam (peace) and Veeram (heroic), both pleasant emotions, in the delta and gamma bands. We also find that Bibhatsam (disgust) and Karunayam (sorrow), both representing unpleasant emotions, formed a distinguishable pair. On noticing the activeness scale, however, Bibhatsam and Karunayam indicate high and mild intensity emotions, respectively, and hence this pair although similar on the pleasant dimension, it is dissimilar on activeness scale. In beta band, Hasyam (comic) and Adbhutam (astonishment) were distinguished from Bibhatsam. Similarly, Bhayanakam and Karunayam formed a distinguishable pair in delta, beta, and gamma bands, indicating high and mild intensity.

**Table 5:** Distinguishable pairs (*p* < 0.001) of $\textit{Rasa}$s in different frequency bands

$\textit{Rasa}$ 1	Discriminated ($\textit{Rasa}$s 2)	Band
Bibhatsam	Santam, Veeram and Karunayam	Delta and Gamma
Bibhatsam	Hasyam and Adbhutam	Beta
Bhayanakam	Karunayam	Delta, Beta and Gamma
Sringaram	All	Delta, Beta and Gamma
Sringaram	except Bibhatsam and Bhayanakam	Theta
Sringaram	Santam and Hasyam	Alpha

## Limitations and future scope

We would like to mention a few limitations of this study. Our results are based on the scalp electrodes, which do not have clearly defined source mapping inside the brain, and therefore we confine the findings in the signal space rather than source space. We used a single set of film clips (corresponding to different $\textit{Rasa}$s) which was selected based on the ranking from a group of participants who confirmed the evoking of these particular emotions. The study could be carried out on more such film clip sets for nine $\textit{Rasa}$s. Hence, this study motivates building a benchmark dataset of audio-visual stimuli corresponding to $\textit{Rasa}$s for EEG studies. We acknowledge that thresholding on *p*-value can also vary based on pairs. However, the main objective of the article is to present the significance of bands by utilizing network features. Hence, future works would have ample opportunity to see the pair-wise differences and similarities in more depth, including the role of network features. The EEG experiment involved only Indian students, hence there is a scope for extending the $\textit{Rasa}$ analysis on different races as well, and explore the similarities and differences (if any) from the results reported in this paper. This research contributes to the pioneering work on Indian $\textit{Rasa}$s, reporting network-based similarity and differences in brain responses collected through EEG.

## Conclusion

In this work, we computed the functional connectivity networks, corresponding to nine $\textit{Rasa}$s, that represented the correlations between the activities of brain regions while a person was watching emotional movie clips. In order to identify distinguishable and indistinguishable pairs of $\textit{Rasa}$s, the network features from the corresponding functional networks were employed for the classification task. Our binary classification result (accuracy) between a given $\textit{Rasa}$ pair, were re-affirmed with a permutation test. The two key findings of our study are as follows:

1. Slow (delta band) and fast (beta and gamma bands) brain waves generated the maximum number of distinguishable pairs.
2. Theta and alpha rhythms exhibited higher number of indistinguishable $\textit{Rasa}$s pairs.

Our classification results also highlighted the role of frequency bands in examining the differences between emotions. We found that the delta, beta, and gamma produced the maximum number of distinguishable pairs, whereas theta and alpha waves resulted in more indistinguishable pairs, for which the classifiers failed to generate discrimination with statistical significance. In addition, to gain interpretability of the obtained two groups of frequency bands, we analyzed network properties and observed that the magnitudes of the delta, beta, and gamma networks were mostly lower than theta and alpha bands.

In the delta band, a pair between Bibhatsam and Santam obtained the maximum accuracy of 87.5% with precision, recall, and f1-score of 0.9, 0.85, and 0.86, respectively. A pair between Bibhatsam and Karunayama showed an accuracy of 85% with precision, recall, and f1-score of 0.88,0.85, and 0.84, respectively, and similar performance was achieved for Bhayanakam and Karunayam. The classification accuracy between Bibhatsam and veeram was 82.5% with precision, recall, and f1-score of 0.88, 0.85, and 0.84, respectively. We obtained a similar relationship as the delta in the gamma band, with the highest accuracy of 87.5% between Bibhatsam and Karunayama with precision, recall, and f1-score, of 0.96,0.8,0.84, respectively. Bibhatasam and Santam showed an accuracy of 85%. In the beta band, we obtained a maximum accuracy of 85% between Bibhatsam and Hasyam. Among all $\textit{Rasa}$s, we obtained a maximum classification accuracy of 97% in the delta band between Sringaram and Adbhutam, followed by beta and gamma bands with 95% and 94% with Raudram and Santam, respectively.

Based on the magnitudes of the network metrics, we observe that the Raudram (for 10 network metrics) and Sringaram (for all network metrics) $\textit{Rasa}$s are the extreme emotions, i.e., one of them has a minimum (maximum) value, while the other has a maximum (minimum) magnitude. The other seven $\textit{Rasa}$s, lie in the range set by these two for all the network metrics. Based on this observation, we approximate a $\textit{Rasa}$ scale, where they are placed next to each other on a one-dimensional line. The ordering goes as Sringaram, Bibhatsam, Bhayanakam, Adbhutam, Veeram, Hasyam, Karunayam, Santam, Raudram, where the last six can interchange their positions depending on the frequency bands.

## Data Availability

The dataset used for the current study will be made available on request.
